# Evidence of a Gastro-Duodenal Effect on Adipose Tissue and Brain Metabolism, Potentially Mediated by Gut–Liver Inflammation: A Study with Positron Emission Tomography and Oral ^18^FDG in Mice

**DOI:** 10.3390/ijms23052659

**Published:** 2022-02-28

**Authors:** Maria Angela Guzzardi, Federica La Rosa, Daniela Campani, Andrea Cacciato Insilla, Monica Nannipieri, Maurizia Rossana Brunetto, Ferruccio Bonino, Patricia Iozzo

**Affiliations:** 1 Institute of Clinical Physiology, National Research Council (CNR), 56124 Pisa, Italy; m.guzzardi@ifc.cnr.it (M.A.G.); larosa.fed@gmail.com (F.L.R.); 2 Department of Surgical, Medical, Molecular Pathology and Critical Care Medicine, Division of Pathology, Pisa University Hospital, 56124 Pisa, Italy; daniela.campani@med.unipi.it (D.C.); andrea.cacciatoinsilla@med.unipi.it (A.C.I.); 3 Department of Clinical and Experimental Medicine, University of Pisa, 56126 Pisa, Italy; monica.nannipieri@med.unipi.it (M.N.); maurizia.brunetto@unipi.it (M.R.B.); 4 Hepatology Unit, Department of Medical Specialties, Laboratory of Molecular Genetics and Pathology of Hepatitis Viruses, Pisa University Hospital, 56124 Pisa, Italy; 5 Institute of Biostructure and Bioimaging (IBB), National Research Council (CNR), 80145 Napoli, Italy; ferruccio.bonino@unipi.it

**Keywords:** intestinal lumped constant, glucose absorption, positron emission tomography, enteric hormones, adipose tissue and cerebral metabolism

## Abstract

Interventions affecting gastrointestinal (GI) physiology suggest that the GI tract plays an important role in modulating the uptake of ingested glucose by body tissues. We aimed at validating the use of positron emission tomography (PET) with oral ^18^FDG administration in mice, and to examine GI effects on glucose metabolism in adipose tissues, brain, heart, muscle, and liver, and interfering actions of oral lipid co-administration. We performed sequential whole-body PET studies in 3 groups of 10 mice, receiving i.p. glucose and ^18^FDG or oral glucose and ^18^FDG ± lipids, to measure tissue glucose uptake (GU) and GI transit, and compute the absorption lumped constant (LCa) as ratio of oral ^18^FDG-to-glucose incremental blood levels. GI and liver histology and circulating hormones were tested to generate explanatory hypothesis. Median LCa was 1.18, constant over time and not significantly affected by lipid co-ingestion. Compared to the i.p. route, the oral route (GI effect) resulted in lower GU rates in adipose tissues and brain, and a greater steatohepatitis score (+17%, *p* = 0.03). Lipid co-administration accelerated GI transit, in relation to the suppression in GIP, GLP1, glucagon, PP, and PYY (GI motility regulators), abolishing GI effects on subcutaneous fat GU. Duodenal crypt size, gastric wall ^18^FDG uptake, and macro-vesicular steatosis were inversely related to adipose tissue GU, and positively associated with liver GU. We conclude that ^18^FDG-PET is a suitable tool to examine the role of the GI tract on glucose transit, absorption, and bio-distribution. The GI effect consists in the suppression of glucose metabolism selectively in organs responsible for energy intake and storage, and is blunted by lipid ingestion. Modulation of gut and liver inflammation, as reflected by high GU, may be involved in the acute signalling of the energy status.

## 1. Introduction

Positron emission tomography with ^18^F-fluorodeoxyglucose (^18^FDG-PET) has proven to be an important tool to describe organ-specific glucose metabolism in vivo, allowing to detect and monitor abnormalities underlying metabolic and cardiovascular diseases in insulin-sensitive tissues [1,2,3,4,5,6,7]. Tissue metabolism has been most commonly studied under standardized euglycemic hyperinsulinemic conditions, in which controlled glucose and insulin infusions are delivered via the intravenous route to maintain equal plasma levels in all study subjects [2,3,4,5,7,8]. Likewise, ^18^FDG is administered intravenously to quantify organ-specific glucose metabolism during the procedure [2,3,4,5,6,7,8]. Notably, this paradigm does not account for the physiological entry of glucose in the body, occurring via oral ingestion and gastrointestinal (GI) absorption. More invasive studies comparing intravenous and oral glucose delivery have emphasized differential effects on insulin secretion due to incretin release [9], and a recent human study shows that the oral (vs. intravenous) route of glucose loading affects lipolysis and systemic glucose clearance [10], though the organs responsible of such action could not be assessed. In addition, the administration of a single glucose or standard meal, passing through the GI tract, has been associated with systemic and liver inflammation [11,12], though it remains to be established whether these effects require specifically the GI entry route. Accumulating evidence from, e.g., bariatric surgery [13,14,15] or drugs affecting GI function (GLP1 agonists [15,16], metformin [17,18,19,20]), point to the GI tract as an important regulator of tissue glucose uptake (GU).

We postulate that PET imaging may allow to investigate—in vivo and non-invasively—the role of the GI tract in modulating tissue specific GU in metabolically relevant organs under physiological conditions of oral meal ingestion. PET imaging of orally ingested ^18^FDG has been tested in oncology patients to overcome venous inaccessibility following chemotherapy [21,22,23], and in healthy rodents [24] to examine drug transit through the GI tract. Despite its feasibility and potential relevance, this technique has not been used to image the fate of ingested glucose in body organs in the context of metabolic or dietary regulation.

The aims of this study were to (1) evaluate feasibility of in vivo ^18^FDG-PET imaging to study GI transit and absorption, and tissue bio-distribution of orally ingested glucose in mice; the main validation point was to define whether ^18^FDG absorption shows a stable and proportional relationship to glucose absorption over time, allowing the absorption lumped constant (LCa) to be computed; (2) define whether the GI tract plays a role in modulating organ-specific GU in subcutaneous, visceral, and brown adipose tissue, myocardium, muscle, liver, and brain, and the relation with hepatic lipid accumulation and inflammation; and (3) examine the effect of lipid ingestion on glucose GI transit and uptake in body tissues. These aims were addressed by comparing sequential PET imaging in mice after intraperitoneal (i.p.) or oral administration of ^18^FDG and glucose, the latter with, and without, lipid co-administration. Circulating enteric and pancreatic hormones, as well as morphological differences in GI crypts and villi, and in the liver were measured to generate explanatory hypotheses.

## 2. Results

**Figure 1:**
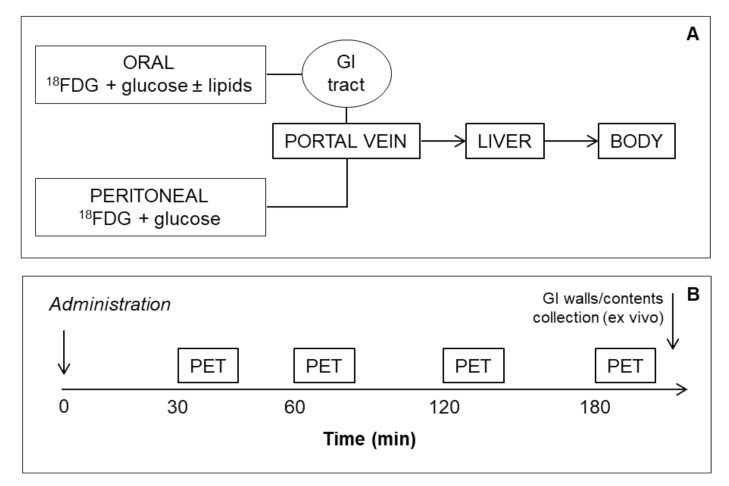
Study design: The design (**A**) involved different types of ^18^FDG and glucose delivery to either by-pass the GI wall (i.p.), or transit across the GI wall (oral glucose, with and without the addition of oral lipids). Panel (**B**) shows the imaging sequence of 4 PET scans, followed by animal sacrifice, activity counting of GI walls and/or GI contents and histological examinations of liver, duodenum, and colon.

Figure 1 summarizes the study concept and design. We stratified *n* = 30, 4 months old B6129SF2J into three groups to receive: (a) i.p. ^18^FDG + glucose (*n* = 10), via i.p. injection; (b) oral ^18^FDG + glucose (*n* = 10), via gavage; and (c) oral ^18^FDG + glucose + intralipid solution (*n* = 10), via gavage. Mice underwent four whole-body 10-minute PET scans with monitoring of glycemia, and then were killed for collection of blood (for plasma incretins, adipokines, fibroblast growth factor, FGF21, appetite regulating, and pancreatic hormones) and GI walls and lumens (stomach, duodenum, jejunum, caecum, and colon) to be counted ex vivo, and analyzed histologically, together with liver samples. No adverse reactions were seen in animals exposed to the procedures. Technical failure in image acquisition occurred in one mouse per oral group, leading to *n* = 10, 9, and 9 analyzed cases.

### 2.1. Gastrointestinal Transit of ^18^FDG

**Figure 2:**
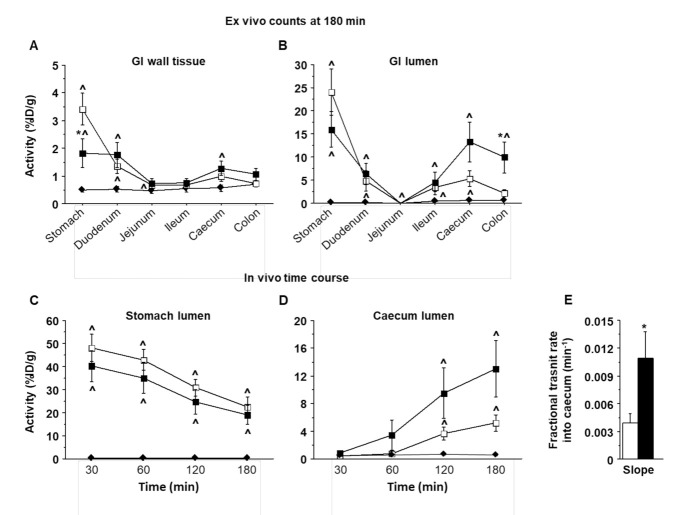
Gastrointestinal activity and transit rate: Top panels show ^18^FDG activity levels, as recovered at the end of each study by ex vivo counting, either in the GI walls (**A**) or in the GI lumens (**B**), showing high levels of ^18^FDG in the gastric wall after ingestion of glucose alone, and high levels of ^18^FDG in distal lumens in the oral glucose + lipid protocol. Bottom panels show in vivo time courses of ^18^FDG activity in the stomach (**C**) and caecum (**D**), as obtained by PET imaging, demonstrating greater tracer levels in the caecum during oral lipid and glucose co-administration than during glucose administration alone, as reflected by a several-fold difference in fractional transit rate constants (**E**), i.e., caecum inward rate constants estimated as slope (absolute activity values). Panels (**A**–**D**) include groups receiving i.p. glucose (black diamond), oral glucose (white square), and oral lipids + glucose (black square); panel (**E**) includes groups receiving oral glucose alone (white bar) or with lipids (black bar). Differences between consecutive walls (**A**,**B**), or changes over time (**C**,**D**) within groups were mostly significant; to limit figure complexity, these comparisons are not shown. * *p* < 0.05 vs. oral glucose alone, ^ *p* < 0.05 vs. i.p. glucose.

Ex vivo ^18^FDG measurements along GI segments (from stomach to duodenum, jejunum, ileum, caecum, and colon) are given in Figure 2A,B, and in vivo ^18^FDG time-courses in the stomach and caecum (30, 60, 120, and 180 min by PET imaging) are shown in Figure 2C,D. Both curves indicate that the amount of tracer reaching distal GI tracts at 180 min was enhanced by the co-administration of lipids compared to glucose alone, suggesting faster GI transit. The slopes of the linear function fitted across caecum ^18^FDG time-course values showed a several-fold increase in transit after lipid co-ingestion compared to glucose alone (Figure 2E). Compared to the i.p. route, oral glucose resulted in significantly greater ^18^FDG extraction in the gastric wall at 180 min (Figure 2A), and this uptake was reduced by lipid co-ingestion.

### 2.2. Lumped Constant

**Figure 3:**
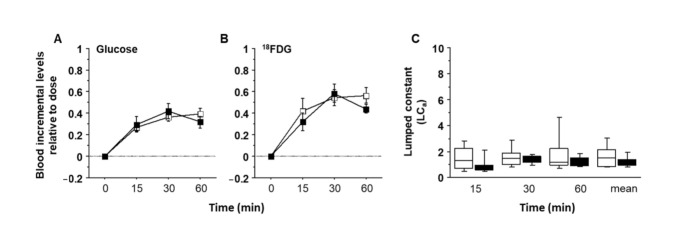
Lumped constant for GI absorption: Time course of dose-normalized increments in blood glucose and ^18^FDG activity levels (**A**,**B**). Panel (**C**) shows stability over time and between groups of the FDG-to-glucose GI absorption ratio, with LCa = 1.18 as median value.

Incremental levels of glucose and ^18^FDG in blood after oral administrations are shown in Figure 3A,B. Trends for glucose and ^18^FDG were similar and unaffected by the type of oral protocol. Their ratio, representing the LCa in Figure 3C documented stable values over time in both oral protocols (i.e., with or without lipids co-administration), with a median value of 1.18, and no significant difference between the two orally administered groups (*p* = 0.51).

### 2.3. Tissue-Specific GU

**Figure 4:**
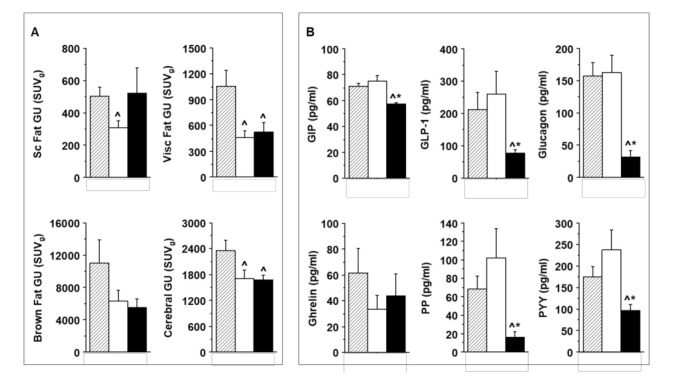
Significant GU rates and entero-pancreatic peptides: Integrated tissue-specific GU rates (**A**), after the oral (white bar) vs. the i.p. route (hatched bar) of glucose delivery, resulting in a reduction in adipose tissues and cerebral GU; the oral co-administration of lipids (black bar) abolished this GI effect in subcutaneous fat. The assessment of hormones involved in GI function and motility, and appetite control (**B**) demonstrated a pronounced suppressive effect of oral lipid administration. * *p* < 0.05 vs. oral glucose alone, ^ *p* ≤ 0.05 vs. i.p. glucose.

By design, similar blood levels of glucose and ^18^FDG in the three study groups were achieved (Figure S1). Significant time-integrated GU results are given in Figure 4A. Compared to the i.p. route, the oral administration of glucose alone resulted in markedly lower GU rates in white adipose tissues, and in the brain (Figure 4A), with no difference observed in muscle, liver, and myocardium (data not shown). The co-administration of lipids abolished the effects caused by oral glucose alone in subcutaneous adipose tissue, but not in the other tissues.

### 2.4. Enteric, Pancreatic, and Fat Hormones

Figure 4B and Figure S2 summarize results concerning plasma hormone and adipokine levels, as determined at the end of the imaging sequence. The oral glucose protocol was characterized by tendencies for greater MCP1, TNFα, PP, PYY, and incretin levels, compared to the other protocols (ns) at 180 min after glucose loading. The co-administration of lipids caused a significant and pronounced, i.e., 30–85% suppression in GIP, GLP1, glucagon, PYY, and PP.

### 2.5. Histology of Duodenum, Colon, and Liver

**Figure 5:**
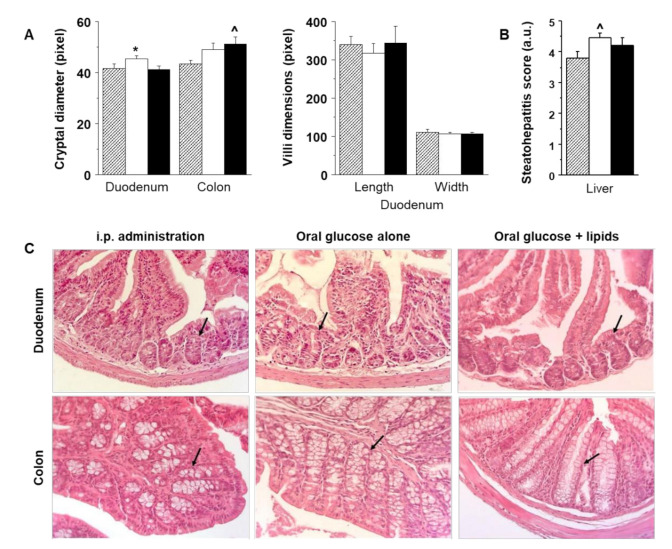
Intestinal villi and crypts, and steatohepatitis score: Histology parameters (**A**), showing greater duodenal crypt dimensions after oral glucose (white bar) than glucose + lipid (black bar), and a progressive enlargement in colon crypts in the oral glucose and glucose + lipid protocols compared to the i.p. group (hatched bar), and corresponding steatohepatitis scores (**B**), showing higher values in the oral glucose protocol compared to the i.p. group. Panel (**C**) provides representative examples of histological sections in the three study groups, in which arrows were positioned to indicate crypts. * *p* < 0.05 vs. oral glucose + lipid, ^ *p* ≤ 0.05 vs. i.p. glucose.

Histological examinations were carried out in 7–9 mice per group, achieving the expected quality. Dimensions of duodenal villi and crypts are shown in Figure 5A,C. The length and diameter of intestinal villi were not different between study groups. Instead, duodenum crypt diameters were greater after glucose ingestion, and colon crypts were larger in oral protocols compared to the i.p. group. Liver histology showed a significantly higher steatohepatitis score after oral than i.p. glucose administration (Figure 5B). As explained in Methods, the steatohepatitis score refers to the cumulative diagnosis, as sum of steatosis grades, lobular inflammation, and ballooning grades, reflecting progressive disease severity stages. Individual components of the score are given in Table 1.

### 2.6. Correlations

Duodenal crypt size was significantly related with villi width (r = +0.46, *p* = 0.047) and tended to be associated with stomach wall GU (Table 2). Both crypt diameter and gastric GU (glucose extraction) were negatively related with GU in subcutaneous, visceral, and brown adipose tissue, and gastric GU was positively associated with liver GU (Table 2). In the oral protocols, gastric GU was additionally related to lower GIP (r = −0.52, *p* = 0.02) and GLP1 (r = −0.42, *p* = 0.07). Colon crypt diameter was positively correlated with ^18^FDG colon content at 180 min (r = +0.45, *p* = 0.029). Hepatic macro-vesicular steatosis was significantly correlated with liver GU (positively), and adipose tissues GU (negatively).

## 3. Discussion

Our study demonstrates that the oral administration of ^18^FDG is a feasible procedure to investigate organ metabolism after oral glucose administration. Our data document that ^18^FDG and glucose GI absorptions are proportional and similar in magnitude. We expressed the intestinal lumped constant term as the relative amount of ^18^FDG vs. glucose appearing in the circulation. The appearance rate in blood was found to be slightly superior for oral ^18^FDG than glucose, with a median value of 1.18 that is comparable to LC values in a variety of other body organs [2,4,8]. Notably, the proportion of orally administered ^18^FDG entering the circulation—as ratio to the respective proportion of glucose—was stable over time and across different glucose levels or during lipid co-ingestion, suggesting that ^18^FDG imaging after oral administration may provide robust information on the fate of glucose under varying metabolic conditions. Our validation paradigm remains to be tested in models of obesity and/or diabetes before the current assumptions can be generalized to metabolic disorders.

This study was designed to detect whether there is a GI effect on GU in metabolically relevant organs. In order to answer this question, we imaged ^18^FDG after i.p. or oral delivery. Both routes are drained into the liver by the portal or mesenteric veins, and therefore the only difference between protocols is that i.p. drainage bypasses the GI tract, whereas the oral route does not. Under this design, any difference observed between the two protocols can be ascribed exclusively to a GI effect. The most important finding of the study was that the GI effect was selective towards GU in adipose tissues and in the brain. The GI effect translated in a reduction in GU in adipose and cerebral tissues compared to i.p. glucose delivery. Interestingly, white fat and brain are the organs regulating energy intake and storage, and glucose represents the main fuel for both tissues, and the lipogenetic drive in adipose tissue. Our data suggest that the sensing of abundant glucose in the upper GI tract (signaling to the liver) may down-regulate energy storage. In fact, we observed an inverse correlation between adipose tissue GU and stomach-wall ^18^FDG uptake or duodenal crypt dimensions and a positive correlation between stomach-wall ^18^FDG uptake and liver GU. ^18^FDG uptake in proximal GI walls may serve as nutritional signal, and duodenal crypts are intensively involved in food digestion, upper GI absorption, and enteric hormone production [25]. We observed correlations between crypt sizes, stomach wall metabolism, and incretins, suggesting that glucose may activate proximal crypts. We also found that steatohepatitis scores were elevated after glucose ingestion compared to i.p. administration, consistent with patterns of circulating TNFα and MCP1. These findings are in line with studies showing that a meal increases liver stiffness and an acute oral glucose load stimulates systemic inflammation [11,12]. We, and others, have previously shown that tissue inflammation may influence GU [26,27,28], which explains the positive correlation between liver GU and macro-vesicular liver fat accumulation. The decline in adipose tissue GU may be partly due to an alleviation of inflammation in this organ [28]. Altogether, the present data suggest that the upper GI wall is mechanistically involved in the metabolic cross-talk occurring between GI and adipose tissues, and that an acute degree of inflammation caused by glucose ingestion may mediate this cross-talk, by stimulating gastric and hepatic GU, in turn signaling to adipose tissues.

The co-ingestion of lipids abolished the suppressive GI effect on subcutaneous fat GU. The redirection of more glucose into subcutaneous fat may be one mechanism whereby lipid intake enhances adipose tissue expansion, regardless of calories, since dietary energy is retained more efficiently in mice fed a high-fat diet than an isocaloric low-fat diet [29]. We tested algorithms to estimate GI transit by using ^18^FDG-PET imaging of GI lumens over time, and observed that the co-ingestion of lipids caused a several-fold acceleration in ^18^FDG transit towards the caecum and colon compared to oral glucose alone. Our mathematically modelled data for GI transit were supported by ex vivo results, showing a 100% group difference in luminal ^18^FDG in the colon at the end of the imaging session. This finding is consistent with the remarkable suppression occurring in the secretion of incretins, glucagon, and appetite regulating hormones, to promote GI motility [30]. As a result, ^18^FDG uptake in the gastric wall and proximal crypt sizes were reduced more by lipid co-administration than by oral glucose alone, whereas colon crypts were enlarged and significantly related with ^18^FDG colon contents. The findings suggest that lipids reduce the release of enteric hormones, accelerating glucose transit (or vice versa), and that cryptal function may be regulated by glucose exposure, both in the duodenum and in the colon. Interestingly, the exclusion of GI walls from food transit (e.g., following bariatric surgery [13,15]) or the use of drugs reducing motility (e.g., GLP1 receptor agonists [15,16,31]) result in an elevation in adipose tissue [13,16] or brain [15,31] GU. In our study, the co-administration of glucose and lipids caused an intermediate situation, with fast transit and low incretin levels, likely balancing brain effects, while stimulating adipose tissue GU.

In the i.p. protocol, we found that a minor portion of ^18^FDG was counter-transported from blood to GI lumen, which confirms previous observations in rodents and humans [14,17]. It remains to be demonstrated whether the leakage of glucose from the circulation, and its putative increase in diseased states (e.g., hyperglycemia) might influence the intestinal milieu and GI function, or whether glucose excretion may become a potential target in disease states, as suggested by observations in bariatric surgery studies [14].

The main limitation of this study is that, due to the need to preserve non-invasiveness in the sequential imaging design, we could not sample blood before the end of the experiments to examine the incretin effect, which is typical of the early absorptive phase. Furthermore, our data apply to normal mice, as studied under acute conditions, and cannot be directly informative on disease or chronic conditions.

In conclusion, ^18^FDG-PET can be used to examine glucose transit and absorption in the GI tract. The LCa was found to be stable over time and in experimental settings. Our results revealed that the GI tract suppresses GU in adipose tissues and brain, and this effect was related to gastric glucose exposure, duodenal crypt dimensions, and liver GU and steatohepatitis scores, which may all signal glucose abundance. A single lipid meal accelerated glucose transit, reduced gastric glucose exposure and duodenal crypt diameters, and directed more glucose into subcutaneous fat. Notably, the involvement of the GI tract in the regulation of GU was specific to the brain and adipose tissue, in which glucose is an essential regulator of cell survival, energy storage, and appetite control. We are tempted to speculate that an energy sensing mechanism is operative in the stomach to protect these fundamental functions, before glucose is absorbed and bio-distributed, and involves an acute inflammatory reaction, relating to GI and liver GU. The protocol proposed has translational value as it can be used in both preclinical and clinical research to understand and modulate (pharmacologically, surgically, or nutritionally) the GI effect and GI transit influencing energy partition, appetite, fat distribution, and microbiota composition in humans and animals.

## 4. Materials and Methods

### 4.1. Study Design

Figure 1 summarizes the study concept and design. We studied *n* = 30, 4-month-old mice (B6129SF2J, The Jackson Laboratory, Bar Harbor, Maine), after an overnight fast. Mice were stratified into 3 groups to receive: (a) i.p. ^18^FDG + glucose (*n* = 10), via i.p. injection; (b) oral ^18^FDG + glucose (*n* = 10), via gavage; and (c) oral ^18^FDG + glucose + intralipid solution (*n* = 10), via gavage. The boluses contained ^18^FDG = 131 ± 6 MBq/kg, glucose = 2 g/kg, intralipid = 0.7 g/kg. Average body weight in the 3 groups was 30.6 ± 1.6 g (ns between groups). Mice underwent 4 whole-body PET scans (IRIS PET/CT small-animal tomograph, Inviscan SAS, France), each lasting 10 min, starting at 30, 60, 120, and 180 min from bolus administration, respectively, under 1–2% (*v*/*v*) isofluorane inhalation (IsoFlo^®^, Abbott Srl, Roma, Italy). To minimize the interference of anesthesia on GI contractility, sedation was not used during inter-scan intervals. Small tail blood samples (1.2 µL) were collected to monitor radioactivity by gamma counter, and glycemia by glucometer. At the end of the experiments, animals were killed by anesthetic overdose, and their abdomen was rapidly accessed for collection of blood and GI and liver specimens. Plasma was used to measure incretins, appetite regulating and pancreatic hormones, adipokines, and FGF21. Radioactivity in progressive GI walls and lumens (stomach, duodenum, jejunum, caecum, and colon) was counted ex vivo using a cross-calibrated gamma counter apparatus. GI and liver tissues were processed for histological examinations. The experimental protocol was notified to the Ministry of Health (Dept. of Public Veterinary Health) in accordance with the D.L.116/92 implementation of directive EEC 609/86 regarding the protection of animals used for experimental and other scientific purposes.

### 4.2. Image Analyses

PET images were reconstructed by a standard OSEM algorithm and corrected for dead time and decay. CT images we reconstructed by FBP algorithm (0.16 × 0.16 × 0.16 mm^3^ cubic voxel size) and standard Ramp Filter. The AMIDE tool (AMIDE-bin 1.0.5) was employed for image co-registration and processing. In one mouse in each oral glucose group, there was a technical scanner failure. Volumes of interest (VOIs) were drawn on images corresponding to the cardiac left ventricular cavity (blood), and tissues, including brain, liver, skeletal muscle, and subcutaneous, visceral, and brown fat, under CT guidance. VOIs were also drawn in the gastric and caecum cavities. Activities in blood, GI cavities, and tissues were normalized to respective administered tracer doses per gram of body weight (%ID/g) at each time point.

### 4.3. Quantification of GI Transit

GI transit was examined by fitting a linear function on time-activity curves of the gastric and caecum cavities obtained in the four PET scans, and evaluating the slope. In addition, we estimated the rate constant for the influx (Ki) of tracer from the proximal to the distal GI tract, by using graphical (Gjedde–Patlak) analysis, in which gastric-activity curves represent the input function and caecum-activity curves the response function. Ex vivo activity counting along GI cavities (stomach, duodenum, jejunum, caecum, and colon) at 180 min was also used to reinforce the interpretation of GI transit data.

### 4.4. Estimation of the LCa

To estimate the lumped constant (LC) for ^18^FDG appearance from the GI into the blood compartment, we calculated the ratio between progressive changes (deltas from baseline) in circulating ^18^FDG and glucose levels, each normalized to the respective administered dose per gram of body weight (*bw*) (Equation (1)):

(1)  [F 18DG (kBq/mLt=n−kBq/mLt=0)F 18DG dose (kBq/grambw)]÷[glucose (μmol/mL t=n−μmol/mLt=0)glucose dose (μmol/grambw)]

Levels appearing in the circulation represent the net outcome of gastrointestinal absorption and peripheral utilization, in which the first component prevails in the first 30–60 min after oral administration. Therefore, the calculation was repeated along the initial 15, 30, and 60 min (=*tn*) time intervals to primarily reflect the absorption LCa. One mouse with negative glucose absorption values was excluded from this analysis.

### 4.5. Quantification of Tissue Glucose Uptake

In tissues, the %ID/g is defined as standardized uptake value (SUV), and reflects the fractional tracer extraction rate constant. SUV values were calculated for all tissues at each scanning time point (30, 60, 120, 180 min = tx), and multiplied by blood glucose levels (average t0–tx) to estimate glucose uptake rates (SUVg) in extra-GI tissues.

### 4.6. Histology

Duodenum and colon biopsies were collected at the time of sacrifice, fixed in 10% neutral-buffered formalin (20–24 h) and processed for paraffin-embedding. From each sample, 5 μm thick sections were cut by microtome, mounted on Polysine™ slides (Menzel-Gläser, Germany, and stained with Hematoxylin–Eosin (HE (Bio-Optica, Milan, Italy), according to standard protocols. Images were acquired for each section at 10× and 40× magnification using an Axioskop optical microscope connected with an AxioCam MRc5 color-camera and AxioVision analysis software (Carl Zeiss, Oberkochen, Germany). Morphological measures were obtained by using the image analysis software ImageJ (version 1.46r, https://imagej.nih.gov/ij). Cryptal diameter was obtained as the mean of 40 Lieberkühn-Galeazzi crypts per sample in both duodenum and colon; the length and diameter of duodenal villi were averaged over 18 villi per sample. Cryptal and villi diameters were assessed at the intermediate point of their full length. Liver samples were fixed in 10% formalin for 24 h, dehydrated, and included in paraffin using the Donatello Diapath automatic tissue processor (Martinengo, Bergamo, Italy), sliced (HistoCore Autocut, Leica BioSystems microtome) with 2 μm thickness, and stained with hematoxylin and eosin using the automated Dako CoverStainer (Santa Clara, CA, USA). Each section was documented at 20× and 40× magnification, by using the Olympus BX51 microscope and connected with an Olympus DP70 digital camera and AnalySIS 5.0 imaging system software (Olympus, Tokyo, Japan). Analyses were adapted from the method of Kleiner et al. [32] to evaluate and score (yes/no, 1/0) vessel dilatation, fibrosis, portal, and lobular inflammation (also graded by foci number at 20× magnification, 1 = one focus, 2 = two-four foci, 3 = >four foci) or ballooning degeneration, and micro/macro-vesicular steatosis (also graded as percentage of affected cells). Cumulative diagnosis (steatohepatitis score) was determined by the sum of steatosis grades, lobular inflammation, and ballooning grades, reflecting progressive disease severity stages.

### 4.7. Biochemical Measurements

Blood was collected at sacrifice in a tube containing EDTA 10%, Foy 5%, Diprotine 10%, and complete mini protease inhibitor cocktail (Merk Life Science S.r.l. Milan, Italy). It was centrifuged for 10 min at 3000 rpm, and plasma was stored at −80 °C. Levels of 14 analytes (GIP, GLP1, PYY, ghrelin, amylin, insulin, c-peptide, glucagon, PP, IL6, TNF-alpha, MCP1, leptin, and resistin) were simultaneously determined by a multi-analyte panel based on Luminex^®^ xMAP^®^ technology (Milliplex map kit, CAT N# MMHMAG-44K, Merk Life Science S.r.l. Milan, Italy).

### 4.8. Statistical Analysis

Data are shown as mean ± sem. Group comparisons were examined by Kruskal–Wallis tests, followed by Mann–Whitney tests. The strength of correlations is expressed by the Spearman coefficient. A *p* value ≤ 0.05 was considered significant.

## Figures and Tables

**Table 1:** Cumulative steatohepatitis score and its components.

	i.p. FDG + Glucose	Oral FDG + Glucose	Oral FDG + Glucose + Lipids
Micro-vesicular fat (% cells)	89 ± 2	86 ± 4	86 ± 3
Macro-vesicular fat (% cells)	5 ± 3	9 ± 4	9 ± 3
Lobular inflammation (yes/no)	0.70 ± 0.15	0.89 ± 0.11	0.78 ± 0.15
Lobular inflammation foci	0.70 ± 0.15	1.11 ± 0.20 ^	0.89 ± 0.20
Cell ballooning (yes/no)	0.10 ± 0.10	0.22 ± 0.17	0.33 ± 0.17
Cumulative steatohepatitis	3.80 ± 0.20	4.44 ± 0.18 *	4.22 ± 0.22

* *p* = 0.03, ^ *p* = 0.1 vs. i.p. group, *n* = 10, 9, and 9, respectively.

**Table 2:** Associations linking GI parameters and organ metabolism.

	Stomach Wall FDG Fractional Extraction		Duodenal Crypt Diameter		Liver Fat Accumulation (Micro)		Liver Fat Accumulation (Macro)	
	r	*p*	r	*p*	r	*p*	r	*p*
Gastric FDG extraction	-	-	+0.49	0.069	-	-	-	-
Sc fat GU	−0.46	0.014	−0.52	0.013	+0.42	0.034	−0.43	0.027
Visc fat GU	−0.52	0.005	−0.56	0.007	-	-	−0.42	0.032
Brown fat GU	−0.43	<0.024	−0.45	0.037	-	-	−0.38	0.054
Liver GU	+0.37	0.051	-	-	−0.45	0.023	+0.47	0.016

Abbreviations: Sc = subcutaneous, Visc = visceral.

## Data Availability

The data presented in this study are available on request from the corresponding author, as they have not yet been uploaded in a public database.

## Supplementary Materials

The following supporting information can be downloaded at: https://www.mdpi.com/article/10.3390/ijms23052659/s1.
