# Supplementary material for: Evidence of a Gastro-Duodenal Effect on Adipose Tissue and Brain Metabolism, Potentially Mediated by Gut–Liver Inflammation: A Study with Positron Emission Tomography and Oral 18FDG in Mice
Source: Int J Mol Sci. 2022 Feb 28;23(5):2659. doi: 10.3390/ijms23052659 (PMC8910830; doi:10.3390/ijms23052659)
Supplement: Supplementary file 1 [file ijms-23-02659-s001.zip › ijms-1575691-supplementary.pdf]

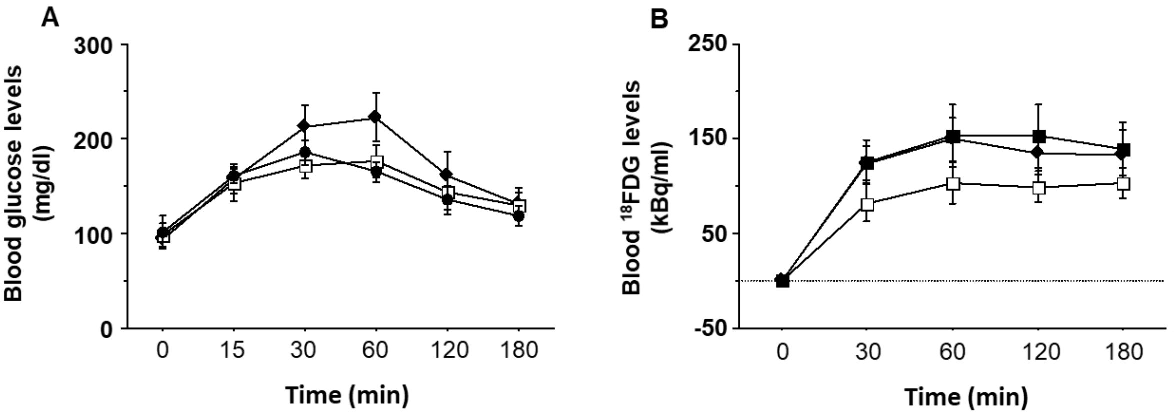

Figure S1. Glucose monitoring. Time course of blood glucose (A) and [18]FDG (B) levels over 3 hours, showing no significant differences between groups (diamonds = i.p., white squares = oral glucose, black squares = oral glucose + lipids administrations), as per design

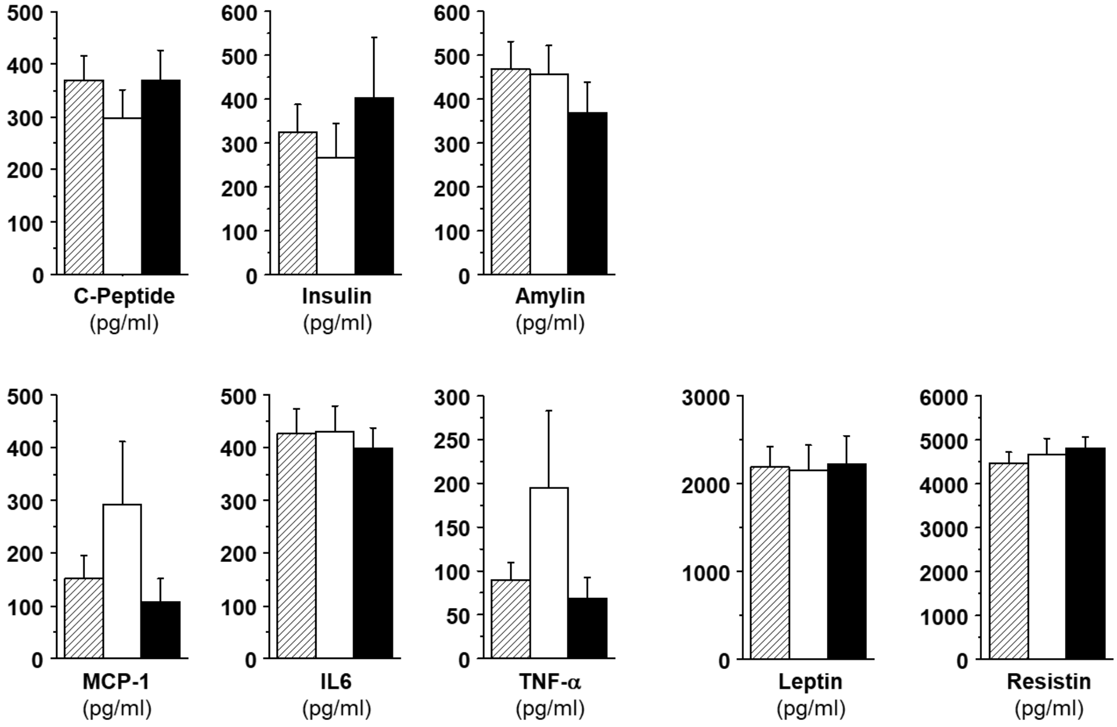

Figure S2. Hormones & cytokines. This figure shows hormones and cytokines that did not show significant group differences after 180 minutes from oral or i.p. administrations. Hatched bar = i.p., white bars = oral glucose, black bars = oral glucose + lipid administrations
